# Immunometabolism features of metabolic deregulation and cancer

**DOI:** 10.1111/jcmm.13977

**Published:** 2018-11-18

**Authors:** Xue Wang, Feng‐Feng Ping, Sahar Bakht, Jingjing Ling, Waseem Hassan

**Affiliations:** ^1^ Wuxi School of Medicine Jiangnan University Wuxi China; ^2^ Wuxi People's Hospital Nanjing Medical University Wuxi China; ^3^ Faculty of Pharmacy and alternative medicine The Islamia University of Bahawalpur Bahawalpur Pakistan; ^4^ Wuxi Children's Hospital Wuxi China; ^5^ Department of Pharmacy COMSATS University Islamabad Lahore Pakistan

**Keywords:** cancer, immunometabolism, metabolic deregulations, tumour microenvironment

## Abstract

Immunometabolism is a branch dealing at the interface of immune functionalities and metabolic regulations. Considered as a bidirectional trafficking, metabolic contents and their precursors bring a considerable change in immune cells signal transductions which as a result affect the metabolic organs and states as an implication. Lipid metabolic ingredients form a major chunk of daily diet and have a proven contribution in immune cells induction, which then undergo metabolic pathway shuffling inside their ownself. Lipid metabolic states activate relevant metabolic pathways inside immune cells that in turn prime appropriate responses to outside environment in various states including lipid metabolic disorders itself and cancers as an extension. Although data on Immunometabolism are still growing, but scientific community need to adjust and readjust according to recent data on given subject. This review attempts to provide current important data on Immunometabolism and consequently its metabolic ramifications. Incumbent data on various lipid metabolic deregulations like obesity, metabolic syndrome, obese asthma and atherosclerosis are analysed. Further, metabolic repercussions on cancers and its immune modalities are also analysed.

## 1 INTRODUCTION

Immune cells are known as a diverse population within the human system that can adopt volatility from their quiescent phase as they get activated by various infections and inflammations. Metabolism is sole source of energy for cells, which they extract from available nutrients in the microenvironment. These cells in turn utilise this energy to perform their vital functions. Interestingly, microenvironment remains virtually same for static cells but obviously keeps changing in case of more dynamic ones such as immune cells which require to circulate to ensure immune surveillance.^1^ The subtle change in microenvironment of immune cells makes them more plastic and adoptable metabolically. The change in metabolic scenario which is ever more consistent for immune cells brings degree of change in their inherent functionalities. Intriguingly immune cells are not only restricted to carry out attack against foreign bodies upon activation but also perform homeostatic roles during normalcy.^2^ Metabolic changes within immune cells are definitely one of the factors for pro‐inflammatory and anti‐inflammatory biases.

Immunity and metabolism are critical determinants of each other as increasing evidence are suggesting the link between these two vital systems of human body.^3^ Even within the immune cells there are distinct metabolic pathways which changes, reshape and adapt according to the given environment. These metabolic pathways within immune cells become vital for their own survival when they traverse to deeper tissues and forced to reside in an environment largely different where they had matured.^1^ The dynamism of immune cells in nature allows the neighbouring cells to readjust their energy demands and hence impact their survival. The same fine‐tuning of energy metabolism in cells in turn regulates the critical functions of immune cells.

## 2 IMMUNOMETABOLISM IN METABOLIC DEREGULATION

**Figure 1:**
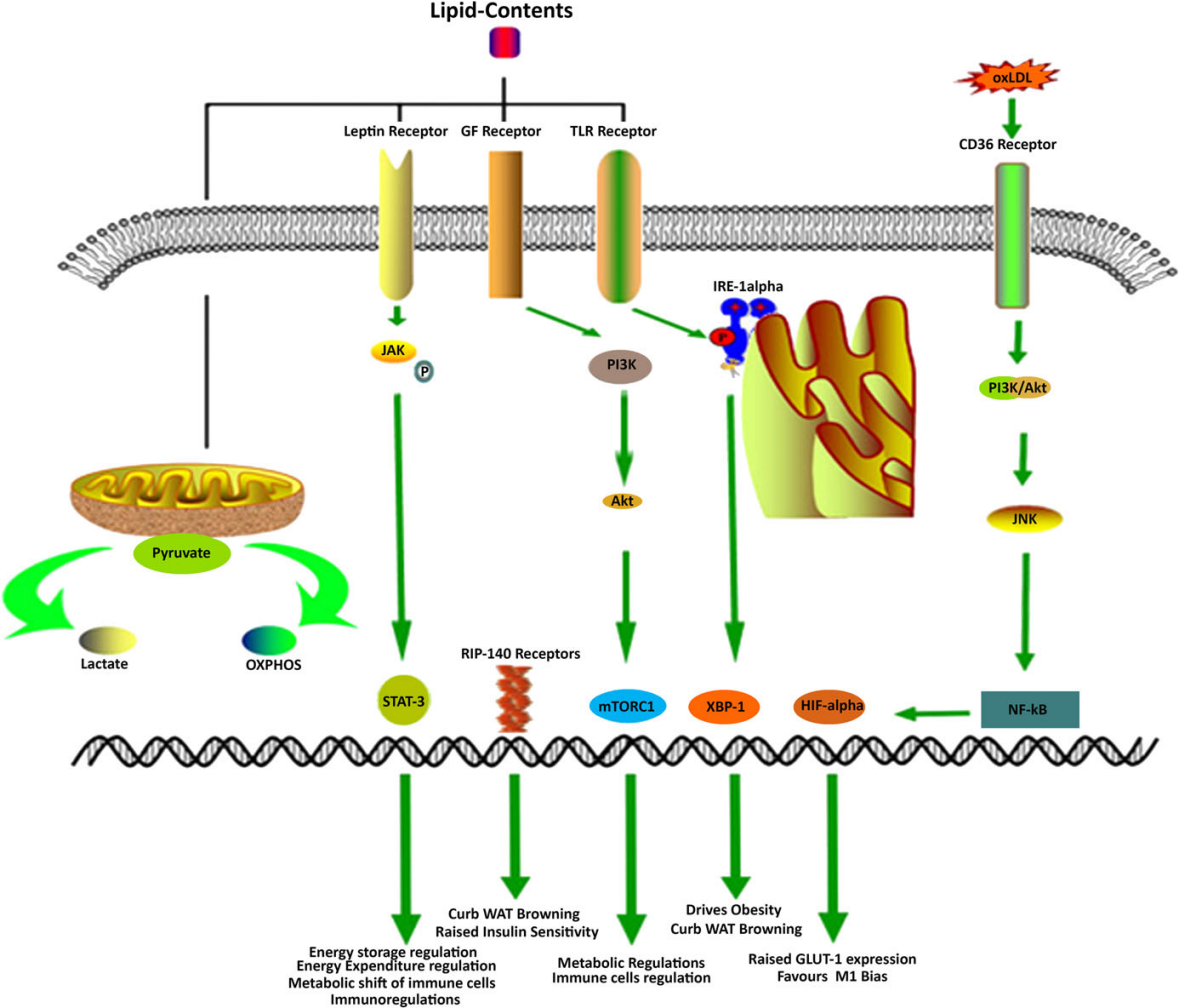
Molecular interactions in Immunometabolism governing lipid metabolic disorders. Induction of Leptin receptor, GF receptors, TLR receptors and CD36 receptors lead to the activation of STAT3, mTORC1, XBP‐1 (through IRE‐1α) and HIF‐1α that steer to interference in lipid metabolism. Additionally, mitochondria also respond to high‐fat contents and divide the pyruvate pathway into lactate and OXPHOS that ultimately decide the inflammatory fate

It has been obviously recognised by the scientific community by now that obesity is an inflammatory state and various immune cells play a pivotal role in accumulating contents for obesity.^4^ Although relationship is well‐established but the basic question of “How” still remain rather obscure. One of the recent pathways in this direction is put forward by Shan et al which recognises inositol‐requiring enzyme 1α (IRE1α) as a “critical switch” mastering M1‐M2 macrophage polarisation and impacting energy homeostasis. Study proves that IRE1α, a sensor of ER stress, ablation in Ern1^f/f^; Lyz2‐Cre mice blocked all kinds of lipid accumulation within body and reversed insulin resistance (Figure 1). Authors also notice the parallel balancing of M1‐M2 bias in white adipose tissue (WAT). Study concluded that IRE1α is responsible for driving obesity and abnormal metabolic deregulations primarily by curbing WAT browning.^5^ Another important observation is put forward by Pu‐Ste Liu et al suggesting the activation of receptor interacting protein 140 (RIP140), present on macrophages, through high‐fat diet (HFD). Receptor Interacting Protein‐140 (RIP140) knockdown in macrophages through transgenic and bone marrow transplantation procedures under HFD induced states remarkably improved WAT browning and improved systemic insulin sensitivity in these mice (Figure 1).^6^ Results were subsequently confirmed by the same group by injecting engineered anti‐inflammatory macrophages (lacking RIP140) marking improvement in insulin sensitivity.^7^ This data hints the presence of specialised metabolic receptors on immune system cells, which ultimately primes its responses according to metabolic states outside. Interesting evidence in this regard is presented recently by Bekkering et al who reported that metabolic signals can induce trained immunity. Series of experiments showed that cholesterol synthesis pathway is essential for training of myeloid cells,^8^ which in turn are implicated in adiposeness and obesity.^9^ Furthermore, innate lymphoid cells (ILC) that are a group of tissue‐resident immune cells have been revealed to exert their vital functions through hosts dietary components and metabolites, which simultaneously affects host metabolism.^10^ In addition an intriguing view is presented by Paula Neto et al^11^ that food additives, which normally are considered harmless, can affect immune system cells that in turn modulates metabolic deregulations like obesity and diabetes. This notion is strengthened by the view that both immune disorders and abnormal metabolic states affect each other in bidirectional way, and more often than not immune activation leads to metabolically abnormal state which may be controlled by epigenetic mechanisms.^12^

Key evidence regarding the metabolic alteration within immune system and its implication on lipid metabolism is provided by Shubham et al recently. Study revealed that arachidonic acid, sphingolipid and glycosphingolipid metabolic pathways were upregulated in visceral adipose tissues (VAT) of non‐alcoholic fatty liver disease (NAFLD) and this phenomenon coexisted with pro‐inflammatory bias in these patients.^13^ It has become widely accepted that macrophages play a pivotal role in lipid metabolism and obesity.^14^ Variety of scientific data are available which implicate pro‐inflammatory M1 macrophages in initiation, worsening and development of various lipid accumulating states.^15^ Current observations have suggested that metabolic reprogramming within macrophages decides their fate and hence their response to environment immediately outside depends on the kind of energy scenario within.^16^ Division and nomenclature of macrophages into M1 and M2 forms represents two different poles of phenotypes that depend on multiple factors like type of ligand for activation of macrophages and nature of microenvironment which resultantly affects intra‐metabolism and hence their varied functions outside.^17^ It has been long established that interferon‐γ (IFNγ) and lipopolysaccharides (LPS) drive the macrophages towards pro‐inflammatory state (M1) and interleukin (IL)‐4 and IL‐13 divert them towards anti‐inflammatory (M2) state.^18^

Metabolic diseases are growing rapidly in present world food scenario. Obesity, NAFLDs, hyperlipidaemia, metabolic syndrome and diabetes are all associated with negative alteration in immune functionalities. Leptin is one of the key hormones secreted from adipose tissues and is involved in regulation energy storage and expenditure.^19^, ^20^ Interestingly leptin has also been linked with the activation of immune system through its receptors. The immunoregulatory role of leptin is surely enhanced under the aforementioned lipid overload states and provides another evidence of immune cells metabolic shift as a result of discrete microenvironmental changes. Leptin receptor (Ob‐R) manifests sequence homology to class I cytokine receptor (gp130) superfamily,^21^ for example, IL‐6, granulocyte colony‐stimulating factor (G‐CSF) and leucocyte inhibitory factor (LIF). It is reported to induce JAK‐STAT, PI3K and MAPK signalling pathways.^22^ Depletion or deficiency of leptin has been reported to cause inefficient phagocytosis and phenotypic abnormalities in macrophages,^23^, ^24^ while alters the production of cytokines from Kupffer Cells (KCs).^25^ Similarly, in dendritic cells leptin is a recognised producer of IL‐8, IL‐12, IL‐6 and TNF‐α, while it reduces MIP‐1‐α secretion.^26^ Likewise, this metabolic hormone affects the functions of mast cells,^27^ neutrophils^28^ and Natural Killer (NK) Cells.^29^ Leptin deficiency is associated with increased susceptibility to damaging effects of TNF‐α and LPS,^30^, ^31^ which may be attributed to the lower number of circulating monocytes in *ob‐ob* mice.^32^ Likewise ob‐ob mice are more prone to infections and have thymic atrophy along with reduced T cells functions.^33^ Leptin has expressed its immunoregulatory effects in human monocytic cells demonstrating 6‐ to 10‐fold increase in secreted IL‐1Ra in a time‐ and dose‐dependent manner. This further exerts that leptin's actions are dependent on functional leptin receptor OB‐Rb present on human monocytes that act through Jak/STAT pathway.^34^ Although correlation between leptin and immune regulation is well‐established but it is uncertain whether these changes are brought about by reorientation of immune cells metabolism. One of the earliest evidence in this direction is provided by Chandra et al^35^ which suggested that malnutrition and starvation induces immunodeficiency, increasing vulnerability to death and infections. mTORC1 is a protein complex that functions as a nutrient, energy and redox sensor. mTORC1 is well credited to be a major metabolic regulator and signalling pathway in immune cells, controlling their responses mediated through metabolic shifts (Figure 1).^36^ Important scientific verification is provided by Mejia et al^37^ linking both leptin and mTORC1 in manipulating immune responses in T cells. Correspondingly, metabolic regulation of T cells and iNKT cells by leptin is demonstrated by Venken et al.^38^

Atherosclerosis is rather more relevant to Immunometabolism because this disease, over the course of decades, have acquired the reputation as a metabolic state as well as immune complication. It's a disease that occurs at the interface of immunity and metabolism and hence become a vital case study in this context.^39^, ^40^ It is well‐established now that metabolic reprogramming of glucose, fatty acids and amino acids in macrophages leads to fatal consequences in atherosclerosis.^41^ Macrophages bind to the activated endothelial wall and transverse to the intimal layer where they expedite the uptake of oxidized low‐density lipoprotein (oxLDL) and orchestrate the synthesis of foam cells. Foam cells along with secreted cytokines generate plaques, which forms the basis of atherosclerosis.^42^ Currently it is appreciated that, so‐called, different forms of macrophages (M1and M2) acquire different routes of metabolism to obtain their energies. M1 follows less efficient metabolic path of glycolysis, which is responsible for the formation of pyruvate and then conversion into lactate via lactate dehydrogenase (LDH), yielding two molecules of ATPs for each glucose molecules. Contrarily, M2 shuttles pyruvate into more conventional mode of metabolism, ie, oxidative phosphorylation (OXPHOS) after passing through krebs cycle (TCA), generating 32 ATP molecules in total.^43^ It is worth mentioning that M1 mode of metabolism causes Warburg metabolic shift, turning the cells more prone to rapid cell division, raising their sustainability in tough metabolic conditions, allowing them to live on lesser metabolic means and hence supporting cancerous growth. Activation of monocytes in response to oxLDL, LPS and hypoxia (all present in atherosclerosis) generates pro‐inflammatory signals inciting NF‐κβ which modulates HIF‐1α that raises the GLUT‐1 expression to increase the uptake of glucose within macrophages (Figure 1).^44^, ^45^ As discussed earlier, pro‐inflammatory M1 choose glycolysis as preferential energy getting path. This scenario allows M1 macrophages to not only pick greater amount of glucose, sapping down host's energy but also serves as a stimulator to secrete more inflammatory cytokines. In one of the classic study Ldlr(−/−) mice were transplanted with bone marrow from HIF‐1α deficient mice within the myeloid cells, which resulted in 72% reduction in atherosclerosis. Similarly HIF‐1α deficient macrophages manifested reduced differentiation to pro‐inflammatory M1 macrophages.^44^ HIF‐1α also facilitates macrophages migration into tissues through activating pyruvate dehydrogenase kinase isozyme 1 (PDK1), which divert the glycolysis towards the formation of lactate (Figure 1).^46^ This proves the imperative contribution of glycolysis in immune cells migration, highlighting the importance of Immunometabolism. Another key study is reported by Bekkering et al^47^ which concluded that short exposure of monocytes to low concentration of oxLDL induces a long‐lasting proatherogenic macrophage phenotype via epigenetic histone changes. Report was further confirmed by Li et al^48^ using nucleolin to protect macrophages from oxLDL induced foam cells synthesis. Further, a detailed and exclusive review on the immunometabolism of atherosclerosis can be read elsewhere.

Another relevant example of Immunometabolism can be traced in obese asthma, which is a state characterised by infiltration of adipose tissue by activated macrophages and mast cells. Periyalil et al in one clinical study demonstrated age and sex specific macrophages activation in obese asthma patients. Study compared obese and non‐obese asthma patients through spirometry, body composition assessment by dual energy X‐ray absorptiometry and found raised levels of sCD163 in former. The study also found elevated plasma CRP and leptin levels in obese asthma adults, while serum tryptase concentrations remained unchanged across different age groups of obese individuals. Finally, it concluded the higher existence of subphenotypes of macrophages in obese asthma patients at the molecular level.^49^ Earlier sCD163 have been positively associated with body mass index (BMI) and type 2 diabetes as well.^50^ A refined study in this context is conducted by Lessard et al which concluded obese asthma patients as different phenotypes, showing poorer asthma control in response to methacholine challenge, when compared with non‐obese asthma patients. Predictably bronchial and systemic inflammatory characteristics were also different in two groups.^51^ Similar evidence were rationalised by others in elegant studies,^52^, ^53^ suggesting a greater culpability of Immunometabolism in this state. Besides, Youssef et al^54^ showed greater involvement of leptin, a metabolic regulator, in obese asthma patients as compared with non‐obese children, manifesting Th1 bias, higher IFN‐γ levels and greater asthma severity. Moreover, the probable role of metabolically induced leptin^55^ and adiponectin^56^ on airway inflammation is increasingly under scientific debate without definite conclusion.^57^ Further, an elegant study is demonstrated by Gibeon et al examining the role of lipid‐laden macrophages on obese asthma patients. Study was performed on 38 asthma patients, 16 patients with chronic cough and 11 healthy control individuals in which lipid‐laden macrophages index (ILLM) was determined. Report deduced the correlation between severity of asthma and higher presence of lipid‐laden macrophages.^58^ Likewise, intriguing scientific observation have been reported linking more devastating actions of mast cells under obese states in asthma patients.^59^ It was observed by Liu et al^60^ that WAT from obese humans and mice have greater mast cells than WAT from their lean counterparts. Conversely mast cell stabilisers were found to be reducing obesity and inflammation concurrently. Conclusion was augmented by Wang et al^61^ and Liu et al^62^. Although mechanism of action by which lipids manipulate the immune responses in asthma and airway inflammation remains largely obscure but few studies have implicated TLR receptors that can trigger the immune activation. For example Zhao et al^63^ decisively showed the involvement of TLR‐4 receptors in airway inflammation following dietary fat induced model.^64^ In addition to the involvement of macrophages and mast cells, the Immunometabolism in neutrophils is also indicated in obese asthma patients.^65^, ^66^ For instance, traditional treatment of asthma responded to lesser degree in obese asthma patients in the presence of neutrophilic airway inflammation.^67^

Another landmark study was reported by Scott et al in which obese and non‐obese adults with asthma, and obese and non‐obese healthy controls were compared by analysing neutrophil percentage and C‐reactive protein level. Sputum neutrophil percentage and severity of neutrophilic asthma was positively found to be correlated with BMI and obesity. In conclusion, saturated and monounsaturated fatty acids were put as vital predictors of neutrophilic airway inflammation in asthma.^68^ Parallel effects of saturated fatty acids were found on lung macrophages along with airways inflammation in mice when fed with high diet.^69^ However it remains debatable that whether obstructive sleep apnoea or asthma is responsible for neutrophilic inflammation in obese cases.^70^, ^71^

Excellent reviews on Immunometabolism are written in current years.^72^, ^73^

## 3 IMMUNOMETABOLISM IN CANCER

Current list of cancer hallmarks is extended by Hanahan et al^74^ with the addition of two critical determinants, ie, deregulating cellular energetics and avoiding immune destruction. The cellular energy reprogramming in tumour microenvironment (TME) shape the response of immune system cells often in such a way that tumour evasion becomes inevitable. Energy metabolism and immune response in TME are so intricately woven that it's tough to predict a obvious winner. The base of complexity lies in the variety of cells that a single tumour may contain. Including cancer cells, fibroblasts, endothelial cells, immune inflammatory cells, adipocytes, extracellular matrix molecules and soluble factors, a tumour mass contain various cellular demands and dynamic energetics.^75^ A recent perspective from Ying Zhang et al has highlighted the metabolic challenges faced by tumour antigen‐specific CD8^+^ tumour‐infiltrating lymphocytes (TILs) in TME which ultimately results in “functional exhaustion” of TILs. This hypoxic and hypoglycaemic environment are sufficient to pose a significant threat to otherwise extremely resilient cells.^76^ More recently, these results were used by Zhang et al by subjecting TILs to extensive metabolically challenged environment which resulted in fatty acid catabolism and PPAR‐α activation.^77^ CD8 and CD4 TILs are previously known to behave differently in hypoxic environment. One of the reports has linked the raised expression of CD137 in TILs when exposed to TME of transplanted and spontaneous mouse tumours, indicating the hypoxic state. Results were confirmed when anti‐CD137 monoclonal antibodies were injected causing liver inflammation to lower and significant achievement of anti‐tumour systemic effects.^78^

Myeloid cells, normally having protective response, transform into their immunosuppressive counterparts due to metabolic reprogramming in TME and termed as myeloid‐derived suppressor cells (MDSC). These cells are known to curtail anti‐tumour T cell functions and shield tumours from the effects of chemotherapy and radiation therapy.^79^ Fokhrul et al explained that the tumour‐infiltrating MDSCs (T‐MDSC) have greater fatty acid uptake and activated fatty acid oxidation (FAO) as compared to their pre‐energy programmed state. It was accompanied by greater mitochondrial mass, upregulation of vital FAO enzymes, and higher oxygen consumption rate. Expectedly, the inhibition of FAO alone greatly reduced the production of inhibitory cytokines and increased the anti‐tumour efficacy.^80^ Similar results were observed in lung cancer^81^ and chronic inflammation,^82^ exerting a point that Immunometabolism in TME plays a pivotal role in functional immune shift from protective actions to tumour supportive roles.

Treg cells are well‐known to possess immunoregulatory activities that inhibit the anti‐tumour responses from immune cells. Wang et al summarised the immunomodulatory functions of Tregs in the TME and discussed the metabolic regulation of Tregs that can facilitate intratumoural Treg storage^83^ and ultimately anti‐tumour responses. Not all the studies have favoured the conventional anti‐tumour role of Tregs within TME. For example, Whiteside have demarcated Tregs into “good” and “bad” ones, the phenomenon, she states, depends on the in situ molecular pathways.^84^

It is now believed that internal metabolism of cancer cells not only helps support the malignant growth but also affects malignant cells phenotype.^85^ The residency of different malignant phenotypes in the presence of dissimilar immune cells within TME is a well reported evidence.^86^ Loss of p‐53 tumour suppressor to contribute in malignant transformation^87^; mutation‐driven metabolic reprogramming through oncometabolites^88^; glucose deprivation^89^ and lactate accumulation^90^ in cancer cells are few of the examples of metabolism induced malignant transformation. The ability of tumour cells to regulate the local nutrients^91^ and waste products of tumour metabolism also play a critical role in determining the fate of metabolic deregulation of cancer.^92^ Moreover, recent emergence of LncRNAs and their metabolic implication in cancers^93^, ^94^, ^95^ provides an interesting dimension to this multi‐headed disease.

Cancer cachexia (CC) is a complex wasting syndrome associated to the majority of cancers, and characterised by abnormal immune cells activation and their presence in metabolic tissues. Increased immune cells presence in WAT of CC patients,^96^ is one of the evidence that how metabolic states in cancer are affected by Immunometabolism. Similar findings were expressed in mice.^97^ Giving a more specific perspective, Rydén et al implicated lipolysis and exonerating inflammation, cell death and lipogenesis in adipose tissue loss in CC. However, study was still able to manifest IL‐6 as a contributing factor.^98^ Results were previously supported by Agustsson et al, indicating adipocyte lipolysis as major factor per se in CC.^99^ Recently, IFN‐γ role in CC^100^ is also appreciated, which is a pleiotropic cytokine that regulates several immune and metabolic functions. These all authentications prove that Immunometabolism plays a vital role and gives subtle dimension to CC. However, Immunometabolism in CC is understudied and further research is required to unravel the mechanism of this open secret.

## 4 CONCLUSION AND FUTURE DIRECTIONS

Based on the above discussion, it is plausible to conclude that molecular metabolic contents play a vital role in priming surrounding immune cells to given conditions by affecting their metabolic pathways. Immune cells metabolic changes, in turn, often lead to metabolic deregulation outside, which culminates into metabolic diseases. Furthermore, tumourigenesis has inflammatory traits that can ultimately lead to metabolic deregulation to worsen the deadly disease known as cancer. Future directions can be traced on the existing gaps in the incumbent data which include but not restricted to (a) knowing the exact mode of actions of immunometabolic states and observing the relevant signal transduction; (b) finding important drug therapy targets within the immunometabolic axis that can be prospectively used to treat metabolic diseases; (c) exploring the natural means to switch or shuffle the immunometabolic pathways in a specific direction in favour of patients.

## CONFLICT OF INTEREST

The authors confirm that there are no conflicts of interest.
